# Cartilage restoration technique of the hip

**DOI:** 10.1093/jhps/hnv061

**Published:** 2015-10-01

**Authors:** Rodrigo Mardones, Catalina Larrain

**Affiliations:** 1. Department of Orthopedics, Head of Tissue Engineering Laboratory; 2. Department of Orthopedics, Clínica Las Condes, Santiago, Chile

## Abstract

Hip cartilage lesions represent a diagnostic challenge and can be an elusive source of pain. Treatment may present difficulties due to localization and spherical form of the joint and is most commonly limited to excision, debridement, thermal chondroplasty and microfractures. This chapter will focus in new technologies to enhance the standard techniques. These new technologies are based in stem cells therapies; as intra-articular injections of expanded mesenchymal stem cells, mononuclear concentrate in a platelet-rich plasma matrix and expanded mesenchymal stem cells seeded in a collagen membrane. This review will discuss the bases, techniques and preliminary results obtained with the use of stem cells for the treatment of hip cartilage lesions.

## HIP CHONDRAL LESIONS

Femoroacetabular impingement (FAI) is frequently associated with chondral damage. The abnormal contact between the femoral neck and the acetabular rim results in labral detachment and acetabular chondral damage [1,2].

In the hip, the types of chondral lesions differ from other joints; the cam type femoroacetabular impingement is frequently associated with chondrolabral junction damage with the subsequent acetabular cartilage detachment [3].

Delamination is a characteristic chondral lesion of the hip (wave sign), in which the cartilage detaches from the subchondral bone leading to a ‘bag lesion’ and chondral flaps. Outerbridge is the most used chondral lesion classification system although delamination was not originally described it could be considered as a Type III. Konan *et al*. [4] recently described a new classification system for hip chondral lesions, including the wave sign, delamination and chondrolabral lesions considering extension and location.

The frequency of chondral lesions in hip arthroscopy for femoroacetabular impingement is high, up to 67.3% of the patients, as described by Nepple *et al*. [5]. Risk factors for the presence of a chondral lesion are: male, tonnis 1 or 2 and alpha angle over 50°.

### Standard treatment for chondral lesions

The arthroscopic treatment of chondral lesions of the hip is limited to excision (rim trimming and femoral neck osteoplasty), debridement, chondroplasty and microfractures. Rim trimming and femoral neck osteoplasty could lead to the complete excision of the chondral lesion if located in the overcoverage area. When the chondral damage extends beyond the resection area, the treatment of choice will be chosen according to Outerbridge or Konan classifications, as follows:

#### Type I or II

The treatment of choice in this type of lesions is thermal chondroplasty. It has shown to be a safe technique for closed chondral lesions leading to morphological changes with better structural characteristics than mechanical debridement [6–9].

#### Delamination

Delamination represents a treatment challenge among chondral lesions. Excising such an area of chondral instability seems an unnecessary surgical maneuver, particularly if the articular cartilage itself may contain a significant number of viable chondrocytes [10]. The main objective is the reattachment of the cartilage to the underlying subchondral bone. This could be achieved with transchondral microfractures, forming an adherent retrolabral clot or with the use of an adhesive such as fibrin glue. Tzaveas and Villar report on a series of 19 patients treated with fibrin adhesive showing improvement in pain and function at 6 months and 1 year after surgery [11].

#### Type III or IV (full thickness chondral lesion)

The indications for microfracture of the hip are similar to the knee and include focal and contained lesions, typically <2–4 cm^2^ in size, (Outerbridge III or IV) including delamination. Microfracture is a marrow-stimulating procedure that brings undifferentiated stem cells from a subchondral perforation into the chondral defect [12]. A clot formed in the microfractured area provides an environment for both pluripotent marrow cells and mesenchymal stem cells to differentiate into stable fibrocartilaginous tissue [13, 14]. Several studies had shown good mid-term results with this technique; however, we know that this fibrocartilaginous tissue does not have the required mechanical properties and eventually will fail, leading to advanced chondral damage and osteoarthritis [14, 15].

### Novel treatments for chondral lesions

As mentioned earlier, the fibrocartilage newly formed at the microfractured area is a low-quality tissue; therefore, we will describe some techniques, based on stem cells therapy, that may lead to a better quality hyaline-like cartilage. The use of these novel technologies has demonstrated promising results in animal and clinical studies [16–19].

## MONONUCLEAR CONCENTRATE (MCC) IN A PLATELET-RICH PLASMA MATRIX

Human platelet-rich plasma (PRP) studies include overuse pathologies (epicondylitis [20], patellar [21] and Achilles [22] tendinopathies), sports medicine (anterior cruciate ligament reconstruction [23–27] and rotator cuff repair [28–31], chondral pathology (osteoarthritis and focal chondral lesions [33–36]), spine [37, 38], trauma (fractures and pseudoarthrosis [39, 40]) and management of skin lesions (acute and chronic [41, 42]). However, the results of the clinical studies are dissimilar, with proven usefulness in epicondylitis, patellar tendinopathy, pseudoarthrosis and chronic wound management. It is likely that interpatient variability and diversity of commercial kits, preparation, implementation and applied concentrations may play an important role in product efficacy, thus influencing the results [43, 44]. Despite this, the studies agree on the anti-inflammatory and procoagulant effect of the PRP.

Hip Chondral lesions traditionally have been handled equal to other joints, with similar results of that obtained at the knee, but the spherical form of the hip, the composition and anatomy of the cartilage and the unique types of chondral lesions (delamination), make the techniques and the results obtained at other joints not replicable at the hip. Hip chondral lesions clinical studies are limited to treatment with microfractures and fibrin clot (fibrin glue) for delamination [45, 46]. These, though they are small clinical series, showed some promising results in localized chondral lesions. Milano *et al*. [47], in a study conducted in sheeps demonstrated that the use of a PRP clot associated with microfractures achieved a complete filling of thechondral lesion with macroscopic, biomechanic and microscopic characteristics similar to normal hyaline cartilage.

Currently, the PRP clot is used as a carrier or membrane carrier of mesenchymal stem cells, a technique that will be explained later.

In summary, the use of a PRP clot in hip chondral lesions has little published evidence; however, the minimal cost and risks of the procedure associated with the promising results obtained in both animal and preliminary clinical studies support its use in the clinical practice of arthroscopic hip surgery.

### Our treatment of choice

**Fig. 1.**
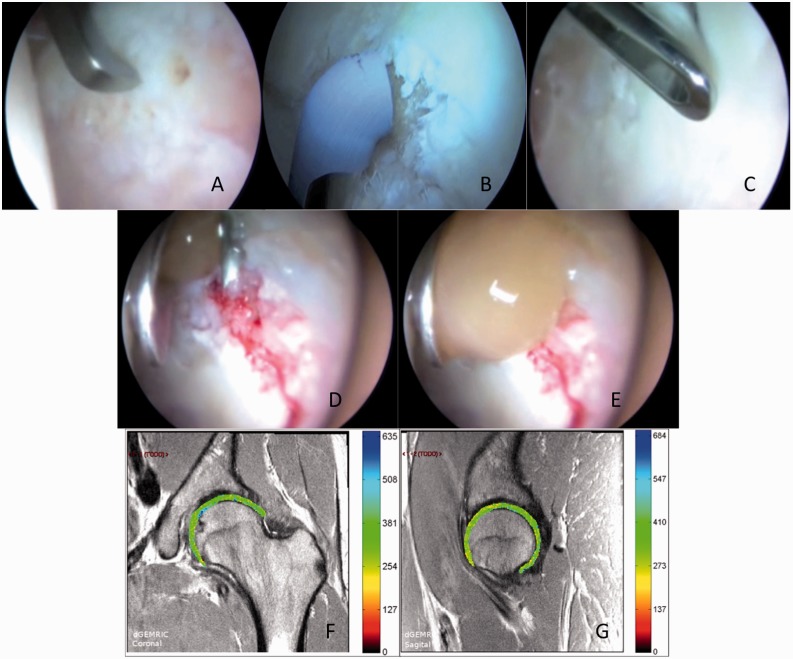
Hip chondral lesions: surgical alternatives and novel technique with platelet-rich plasma and mononuclear cells concentrate. (**A–C**): Standard alternatives for hip chondral lesions. **(A)** Microfractures. (**B**) Thermal chondroplasty. **(C)** Chondral flaps resection. (**D**) and (**E**): Novel surgical technique. The PRP clot is positioned over the microfractured area and mononuclear cells concentrate is instilled under it. (**F**) and (**G**): dGEMRIC images at 6 months postop of the same patient, in which an homogeneus captation of gadolinium is observed meaning the restoration of glycosaminoglican content.

In our clinical practice, the treatment of choice for hip chondral lesions is the use of a platelet-rich plasma clot and MCC over the microfractured area (Fig. 1). The surgical technique is described later.

We have obtained excellent results with this technique, confirming the restoration of glycosaminoglycan concentration by magnetic resonance images (MRI) metabolic type delayed enhanced magnetic resonance imaging gadolium of cartilage (dGEMRIC) (Fig. 1F and G).

#### Surgical technique

After rim trimming and labrum refixation; cartilage assessment is made. If chondral lesion exists, we proceed to harvest autologous bone marrow stem cells, which are centrifugated obtaining 2–4 cm^3^ of autologous bone marrow-mesenchymal stem cells concentrate (average 14 millions of nucleated cells/cm^3^). At the same time, 50 cm^3^ of peripheral blood is taken and centrifugated twice, to obtain 4 cm^3^ of PRP (6 to 9 ×), ready to be activated with autologous thrombin. Treatment of chondral lesion is made as described by Steadman in the knee, with debridement of all remaining unstable cartilage, followed for the removal of the calcified plate. After preparation of the bed, multiple holes in the exposed subchondral bone plate are made, leaving about 3–4 mm between each. Once microfracture is complete, traction is release and we focus on the femoral osteoplasty, obtaining free range of motion with no abnormal contact between acetabular rim and femoral neck–head junction. At the end of the procedure, traction is reinstalled and we proceed to the final part of the procedure. After activation of platelet-rich plasma and clot formation, a slotted cannula is inserted via the anterior portal. Platelet-rich plasma clot is inserted through the cannula and positioned over the microfractured area. A 21-gauge trocar is then inserted passing through previously located clot and autologous bone marrow-mesenchymal stem cells concentrate is instilled under PRP clot. Traction is then released and the procedure is finished.

#### Rehabilitation protocol

Passive motion device is maintained for 8 h. Two crutches with partial weight bearing are indicated for 6–8 weeks. Progressive physical activities are allowed.

### Preliminary results

At the time, 13 patients with chondral lesion of the hip had been treated with microfractures and autologous bone marrow-mesenchymal stem cells concentrate transplanted on a platelet-rich plasma clot. All patients’ symptoms improved over the follow-up period of 8 months (4–12 months). Average hip outcome, Vail Hip and Modified Harris Hip Scores for all patients showed significant improvement at 3 and 6 months. dGEMRIC of 4 patients at 6 months post-operatively revealed complete defect fill and complete surface congruity with native cartilage.

## INTRA-ARTICULAR INJECTIONS OF EXPANDED MESENCHYMAL STEM CELLS

Osteoarthritis (OA) is the most common type of arthritis and the leading cause of disability in the United States [48]. Several systemic treatments, mostly symptom-modifying rather than disease-modifying agents, are available for OA [49]. However, there is a real need for effective, safe, disease-modifying OA therapies that can not only effectively treat those with established OA but also possibly delay or prevent progression in those with early OA [50]. As we mentioned before, in focal chondral lesions, mesenchymal stem cells represent a valid alternative for treatment but multiple chondral lesions or established osteoarthritis are not suitable for focal treatment.

Adult mesenchymal stem cells were originally believed to only differentiate into tissue-specific cells. However, these cells have two major properties that could explain some of the results seen with the intra-articular (IA) injections of expanded mesenchymal stem cells, and this are Homing and Response to specific signals. Homing is a particular property of these cells, meaning that they respond to systemic stimuli and ‘travel to the place that needs repair’. The Homing effect has been demonstrated in several animal studies, using labeled mesenchymal stem cells administered via systemic intravascular route or by direct local implantation, showing the presence of the marker at the injury site [51]. Mesenchymal stem cells have the ability to differentiate into a different tissue in response to specific signals released by the injury site, such as chondrogenic lineage in an osteoarthritic joint [52].

Hip extensive damage or mild OA is usually treated with local infiltrations, symptom-modifying treatments, pain killers and finally a total hip replacement but an increasing number of active patients seek for a non-arthroplasty treatment and stem cells may present as an alternative to this group of patients. IA injections of expanded mesenchymal stem cells have not been described in the hip joint; however, there are some animal and clinical studies in other joints. Mokbel *et al*. [52] labeled autologous adult stem cells suspended in hyaluronic acid were injected intra-articularly into carpal joints in an experimental arthritis induced by IA Amphotericin-B in donkeys. Significant improvement was noted in clinical and radiographic OA and significantly lesser histopathological changes of OA were seen in carpal joints that received stem cells compared with control contralateral joints. Importantly, injected stem cells were incorporated into the articular cartilage of the injected joint, as evident by their integration in the surface of the cartilage and also the interior of the cartilage [52]. Emadedin *et al*. [53] injected expanded mesenchymal stem cells in 6 female patients with OA that required joint replacement. At 12-months follow-up, there was a significant decrease in mean pain, as well as improvements in joint functioning, walking distance, time to gelling, patellar crepitus and knee flexion. MRI obtained at 6 months after treatment showed an increase in cartilage thickness and extension of the repair tissue over the subchondral bone in half of the patients; in addition to a decrease in subchondral bone edema [53]. McIlwraith *et al.* [54] evaluated IA injection of bone marrow-derived mesenchymal stem cells to augment healing with microfractures in horses. At 6 months, arthroscopic and gross evaluation confirmed a significant increase in repair tissue firmness and a trend for better overall repair tissue quality in treated joints compared with microfractures alone. Immunohistochemical analysis showed significantly greater levels of aggrecan in repair tissue treated with stem cells injection [54].

In summary, the use of IA injections of expanded mesenchymal stem cells in OA has little published evidence; however, in a young active patient it seems to be a promising non-arthroplasty treatment.

### Our treatment of choice

In our clinical practice, the treatment of choice for hip diffuse chondral damage and mild osteoarthritis in an active patient seeking for a non-arthroplasty treatment are IA injections of expanded mesenchymal stem cells. The surgical technique is described later.

We have obtained excellent results with this technique, with an increase in hip functional scores (Vail-10 hip score and Harris Hip Score) and a decrease in mean pain values.

#### Surgical technique

**Fig. 2.**
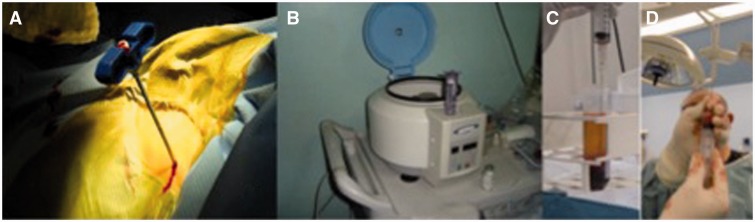
Autologous mesenchymal stem cells concentrate: (**A)** Autologous bone marrow autograft. **(B**) Centrifugation process with a single spin. **(C**) Layer separation by a density filter and identification of mononuclear cells layer. **(D**) Autologous mononuclear cells concentrate-final view.

**Fig. 3.**
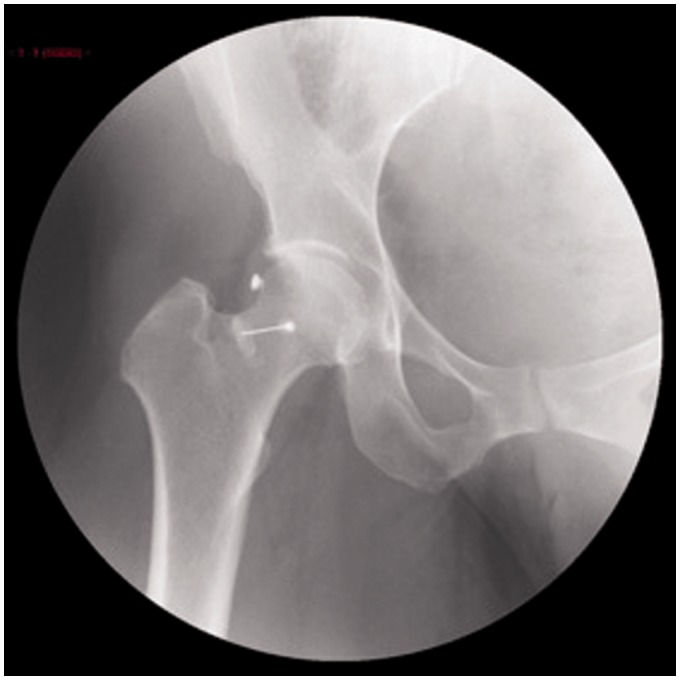
IA injection of expanded mesenchymal stem cells. Fluoroscopic image.

Patients were placed on an operating table in the prone position under general anesthesia. We proceed to harvest 15 cm^3^ of bone marrow from iliac crest and centrifuged obtaining 2–4 cm^3^ of autologous bone marrow-mesenchymal stem cells concentrate (average 14 millions of nucleated cells/cm^3^) (Fig. 2). Bone marrow concentrate is then processed in our GMP laboratory and over a 1-month period, mesenchymal stem cells are expanded to 20 × 10^6^ cells and taken to the hospital in a portable incubator. Under fluoroscopy, cells were injected into the patients’ hips. (Fig. 3)

### Preliminary results

At the time, 7 patients with mild OA of the hip had been treated with IA injections of expanded mesenchymal stem cells. All patients’ symptoms improved over the follow-up period of 10 months (8–14 months). Average Vail-10 and Modified Harris Hip Scores for all patients showed significant improvement at 3 and 6 months. None of the patients has required a total hip replacement at the time.

## EXPANDED MESENCHYMAL STEM CELLS SEEDED IN A COLLAGEN MEMBRANE

Hip chondral lesions can be an elusive source of pain and their treatment is limited to chondroplasty and debridement in partial defects and microfractures for full-thickness chondral lesions. Microfracture involves penetration of the subchondral bone to release blood and bone marrow into the defect, initiating cartilage repair. This technique has produced good clinical results in defects <2 cm^2^. For larger lesions, bone marrow concentrate in a PRP clot seems to be a good alternative. Other treatment options include autologous chondrocyte implantation (ACI), matrix-induced ACI (MACI), autologous matrix-induced chondrogenesis (AMIC) and membrane seeded with expanded mesenchymal stem cells.

ACI has been used increasingly for the repair of larger chondral lesions in the knee. For hip chondral lesion management, only two reports were found. Fontana *et al*. [55] described a case control study in 30 patients with hip chondral lesions, 15 treated with ACI and 15 with debridement alone. At 74-months follow-up Harris Hip Score was significantly better in ACI group compared with the debridement group [55]. Akimau *et al*. [56] described a case of severe chondrolysis and osteonecrosis of the femoral head after a severe fracture dislocation in a 31-year-old man. Twenty-one months after the injury they performed a MACI technique. At 1-year follow-up the subjective hip score and range of motion had increased compared with preoperative values. At 15-months follow-up, biopsy demonstrated a 2 mm thickness cartilage well populated with viable cells and integrated with the underlying bone [56].

Fontana [57] described a fully arthroscopic technique for the hip for AMIC. This is a low cost, single procedure and arthroscopic technique in which the author used a collagen matrix (Chondro-Gide, Geistlich Pharma AG, Wolhusen, Switzerland) over the microfractured area, containing the blood and bone marrow for a better quality reparative tissue [57]. To our knowledge, there is no published clinical data available.

The use of membranes seeded with expanded mesenchymal stem cells has risen in response of some problems observed with the use of MACI, such as the morbility of the donor site and insufficient coverage of the defect area due to some shrinkage effect. This technique has shown good results in other joints but it has not been described in the hip joint.

### Summary

Hip chondral lesions are a frequent finding in hip arthroscopy for FAI. Treatment is often difficult and insufficient. Novel strategies based on cell regenerative therapy represent a promising treatment alternative. We reviewed the novel alternatives for hip chondral lesions treatment and its preliminary results. 

## CONFLICT OF INTEREST STATEMENT

The corresponding author (RM) received fees and royalties related with this research.
